# Association between Thyroid-Stimulating Hormone Level after Total Thyroidectomy and Hypercholesterolemia in Female Patients with Differentiated Thyroid Cancer: A Retrospective Study

**DOI:** 10.3390/jcm8081106

**Published:** 2019-07-25

**Authors:** Young Ki Lee, Hokyou Lee, Seunghee Han, Hyein Jung, Dong Yeob Shin, Kee-Hyun Nam, Woong Youn Chung, Eun Jig Lee

**Affiliations:** 1 Center for Thyroid Cancer, National Cancer Center, Goyang 10408, Korea; 2 Department of Preventive Medicine, College of Medicine, Yonsei University, Seoul 03722, Korea; 3 Division of Endocrinology and Metabolism, Department of Internal Medicine, Hallym Hospital, Incheon 21079, Korea; 4 Division of Endocrinology and Metabolism, Department of Internal Medicine, College of Medicine, Yonsei University, Seoul 03722, Korea; 5 Division of Thyroid and Endocrine Surgery, Department of Surgery, College of Medicine, Yonsei University, Seoul 03722, Korea

**Keywords:** differentiated thyroid cancer, total thyroidectomy, thyroid stimulating hormone, cholesterol, hypercholesterolemia

## Abstract

Less-intense TSH suppression strategies can be used for differentiated thyroid cancer (DTC) patients with a low recurrence risk, but their metabolic outcomes are not well known. We aimed to evaluate changes in the serum cholesterol levels and the risk of hypercholesterolemia according to postoperative TSH levels in 1092 female DTC patients receiving levothyroxine after total thyroidectomy. The preoperative-to-follow-up change in total cholesterol (TC) levels in the TSH level <0.03, 0.03–0.3, 0.3–2, and 2–5 mIU/L groups was −3.69 mg/dL (*p* = 0.006), +0.13 mg/dL (*p* = 0.926), +12.46 mg/dL (*p* < 0.001), and +16.46 mg/dL (*p* < 0.001), respectively. When compared with TSH levels of 0.03–0.3 mIU/L, those of 0.3–2 mIU/L were found to be associated with hypercholesterolemia (adjusted odds ratio (AOR) = 1.86 and 5.08 for TC 200–240 and ≥240 vs. <200 mg/dL) and hyper-low-density lipoprotein (LDL)-cholesterolemia (AOR = 2.76 for LDL-cholesterol ≥160 vs. <130 mg/dL). Additionally, TSH levels of 2–5 mIU/dL were associated with hypercholesterolemia (AOR = 2.85 and 6.95 for TC 200–240 and ≥240 vs. <200 mg/dL) and hyper-LDL-cholesterolemia (AOR = 2.08 and 4.17 for LDL-cholesterol 130–159 and ≥160 mg/dL vs. <130 mg/dL). In patients with normal TSH level maintenance following thyroidectomy, TC levels markedly increased, resulting in an increased hypercholesterolemia prevalence. Metabolic derangement risk due to insufficient levothyroxine replacement should be considered in the adoption of less-intense TSH suppression strategies, postoperatively, in DTC patients.

## 1. Introduction

The incidence of thyroid cancer has rapidly increased over the past few decades globally, and the disease is now the most prevalent type of endocrine cancer [1,2]. Therapies aimed at thyroid-stimulating hormone (TSH) suppression using exogenous levothyroxine are the mainstays in the postoperative treatment of the inhibition of residual tumor tissue growth in patients with differentiated thyroid carcinoma (DTC) [2]. Recommendations for the ideal TSH suppression intensity have changed over time, and current management guidelines suggest the maintenance of a low-normal TSH level range rather than a suppressed TSH level in patients with a low recurrence risk, because intensive TSH suppression had no established benefit in this patient group [3,4].

However, the metabolic outcomes of these less-intense TSH suppression strategies are not well known. Notably, recent studies showed that patients with hypothyroidism had hypercholesterolemia more frequently despite maintaining normal serum TSH levels with levothyroxine replacement therapy, indicating that normal TSH levels did not ensure sufficient thyroid hormone replacement in patients with hypothyroidism [5,6]. Moreover, some studies on DTC patients have suggested that thyroidectomized DTC patients with mildly suppressed TSH levels were the closest to having a physiologic euthyroid status, whereas those with normal TSH levels had a hypothyroid status due to relative triiodothyronine (T3) deficiency driven by the levothyroxine replacement therapy administered to athyreotic patients [7,8]. This evidence raises the concern that the maintenance of normal TSH levels in thyroidectomized patients may increase the risk of hypercholesterolemia associated with insufficient replacement of thyroid hormones. Therefore, we aimed to evaluate changes in the serum cholesterol levels and risk of hypercholesterolemia according to postoperative TSH levels in DTC patients receiving levothyroxine after total thyroidectomy.

## 2. Materials and Methods

### 2.1. Study Participants

We reviewed 1841 women with DTC, who underwent total thyroidectomy from November 2005 to December 2015 aged 19–79 years and were followed-up with lipoprotein profile tests at 1–4 years after thyroidectomy. We excluded those with preoperative serum total cholesterol levels ≥240 mg/dL or missing values, triglyceride levels ≥400 mg/dL at follow-up, TSH levels ≥5 mIU/L or missing values at follow-up, history of high-dose glucocorticoid use within a year and the use of lipid-lowering agents, i.e., statins, ezetimibe, fibric acid derivatives, and omega-3 fatty acids, and those who had undergone radioactive iodine scanning within a year (Figure S1). A total of 1092 women were finally analyzed. The study protocol was approved by the Institutional Review Board of Severance Hospital, Seoul, Korea (IRB number 4-2019-0407; approved on 20 June 2019), and the requirement for informed consent was waived.

### 2.2. Data and Measurements

We obtained the earliest available follow-up medical records, including data on TSH levels and lipoprotein profile at least one year after total thyroidectomy. Data on demographics, medical and social history, and anthropometric measurements were obtained from all participants at follow-up. Serum TSH and free thyroxine levels were measured by chemiluminescent microparticle immunoassay (Architect System, Abbott Ireland Diagnostic Division, Lisnamuck, Longford, Co. Longford, Ireland; the manufacturer’s reference ranges are 0.35–4.94 mIU/L for TSH and 0.7–1.48 ng/dL for fT4) at baseline and follow-up. Lipid profile tests, including those for total cholesterol, triglyceride, and high-density lipoprotein (HDL)-cholesterol, were conducted at follow-up by a routine Hitachi 7600 autoanalyzer (Hitachi Instruments Service, Tokyo, Japan; the reference ranges are 142–240 mg/dL for total cholesterol, 48–200 mg/dL for triglyceride, and 40–75 mg/dL for HDL-cholesterol). Low-density lipoprotein (LDL)-cholesterol levels were calculated using the Friedewald formula [9]. The cutoffs for total cholesterol (200–239 and ≥240 mg/dL) and LDL-cholesterol (130–159 and ≥160 mg/dL) followed the National Cholesterol Education Program Adult Treatment Panel III (NCEP/ATP III) and the Korean Guidelines for the Management of Dyslipidemia criteria [10,11].

### 2.3. Statistical Analyses

Participants were categorized into four groups according to their serum TSH levels at follow-up (<0.03, 0.03–0.3, 0.3–2, and 2–5 mIU/L). Continuous variables were reported as mean ± standard deviation or median (interquartile range). Categorical variables were reported as frequencies and percentages. Intergroup comparisons were performed by Student’s t test or analysis of variance for continuous variables, and a chi-squared test for categorical variables. Comparison of the preoperative and follow-up total cholesterol values was performed by a paired t test. Least-square means of Δ total cholesterol, adjusted for age at follow-up, duration of follow-up, and preoperative cholesterol level, were calculated and compared across the TSH group by analysis of covariance. The association between follow-up TSH level (continuous) and preoperative-to-follow-up changes (Δ) in the total cholesterol was tested by multivariable linear regression models adjusted for age, follow-up duration, body mass index (BMI, kg/m^2^), systolic blood pressure (SBP, mmHg), fasting glucose level (mg/dL), cigarette smoking status (ever vs. never), and preoperative total cholesterol level (mg/dL). Associations of follow-up TSH levels with hypercholesterolemia (total cholesterol level of 200–239 and ≥240 mg/dL) and hyper-LDL-cholesterolemia (LDL-cholesterol level of 130–159 and ≥160 mg/dL) were assessed by multinomial multivariable logistic regression models adjusted for the aforementioned variables. Logistic regressions were further stratified by subgroups of age (<50 or ≥50 years), BMI (<25 or ≥25 kg/m^2^), diabetes presence, and preoperative cholesterol level (<200 or ≥200 mg/dL). Subgroup analyses and tests for interactions were performed against ordinal TSH levels. Finally, as a sensitivity analysis, we repeated our regressions with a conventional logistic model for hypercholesterolemia as binary outcomes (total cholesterol level ≥200 and ≥240 mg/dL; LDL-cholesterol level ≥130 and ≥160 mg/dL). All tests were two-sided, and *p* < 0.05 was considered statistically significant. All analyses were performed using R version 3.4.4 (R Foundation for Statistical Computing, Vienna, Austria).

## 3. Results

### 3.1. Demographics and Descriptive Statistics

The median age of the patients at surgery was 51 (43–58) years, and the median age at follow-up was 53 (45–60) years. The median duration after surgery was 2 (1.4–3.1) years. All patients received levothyroxine for the suppression of TSH levels and/or replacement of the thyroid hormone. At follow-up, 473 (43.3%) patients had TSH levels <0.03; 394 (36.1%), 0.03–0.3; 164 (15%), 0.3–2; and 61 (5.6%), 2–5 mIU/L. The descriptive statistics stratified by TSH group are summarized in Table 1. The groups with low TSH levels below 0.3 mIU/L had a higher proportion of older participants, who underwent earlier follow-up lipid profile tests, compared to the higher TSH level groups.

### 3.2. TSH and Total Cholesterol Level before and after Thyroidectomy

**Figure 1:**
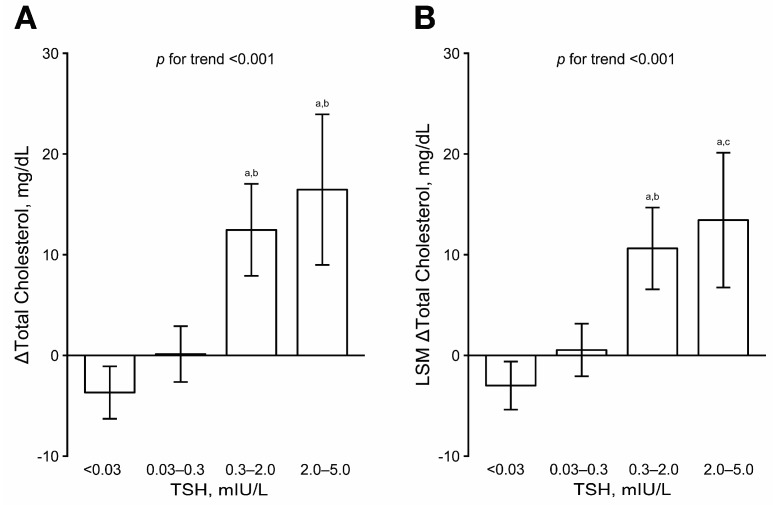
Preoperative-to-follow-up changes (Δ) in total cholesterol level according to TSH group. (**A**) Unadjusted means with intergroup comparisons by analysis of variance and Tukey’s test. (**B**) Least-square means, adjusted for age, follow-up duration, and preoperative cholesterol level, with intergroup comparisons by analysis of covariance and Tukey’s test. ^a^ *p* < 0.001 vs. TSH < 0.03 mIU/L; ^b^ *p* < 0.001 vs. TSH 0.03–0.3 mIU/L; ^c^ *p* < 0.01 vs. TSH 0.03–0.3 mIU/L. LSM, least-square mean; TSH, thyroid-stimulating hormone.

The preoperative TSH levels were comparable between the groups (Table 1). The lower postoperative TSH level groups had higher preoperative cholesterol levels than the higher TSH level groups. At postoperative follow-up, patients with higher TSH levels exhibited significantly higher levels of total and LDL-cholesterol. Relative to the preoperative level, the total cholesterol levels remained unchanged in patients with postoperative TSH levels of 0.03–0.3 mIU/L, but decreased in those with TSH levels <0.03 mIU/L and markedly increased in those with TSH levels greater than 0.3 mIU/L after surgery (Figure 1A). The Δ total cholesterol levels were −3.69 mg/dL (*p* = 0.006), +0.13 mg/dL (*p* = 0.926), +12.46 mg/dL (*p* < 0.001), and +16.46 mg/dL (*p* < 0.001) in the TSH level <0.03, 0.03–0.3, 0.3–2, and 2–5 mIU/L groups, respectively (*p* values calculated by a paired t test). After adjusting for age at follow-up, follow-up duration, and preoperative total cholesterol levels, the least-square means of Δ total cholesterol were −3.00, +0.54, +10.62, and +13.44 mg/dL in the TSH level <0.03, 0.03–0.3, 0.3–2.0, and 2–5 mIU/L groups, respectively (Figure 1B). In the multivariable linear regression, Δ total cholesterol was significantly associated with TSH levels at follow-up after adjusting for age, follow-up duration, and preoperative total cholesterol level (Table 2). The association remained significant after further adjusting for other possible confounders (Table 2).

### 3.3. Association between TSH Level and Hypercholesterolemia after Thyroidectomy

**Figure 2:**
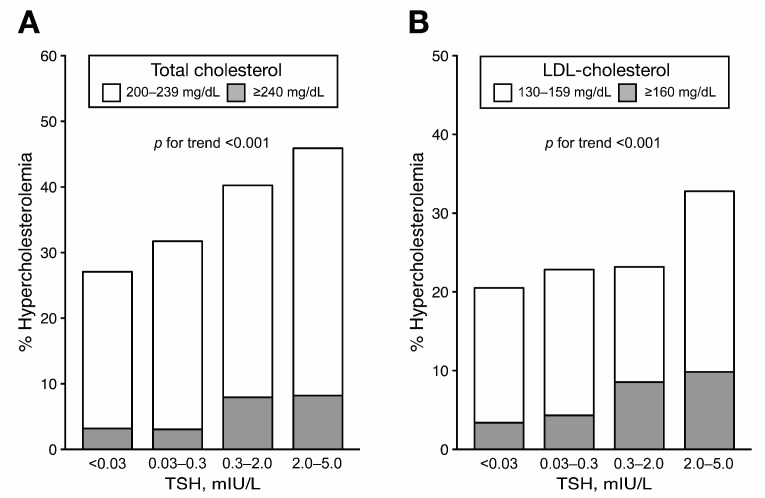
Prevalence of hypercholesterolemia at follow-up according to TSH group. (**A**) Prevalence of hypercholesterolemia defined as total cholesterol level of 200–239 and ≥240 mg/dL. (**B**) Prevalence of hypercholesterolemia defined as LDL-cholesterol level of 130–159 and ≥160 mg/dL. *p* for trend by Mantel-Haenszel test. LDL, low-density lipoprotein; TSH, thyroid-stimulating hormone.

**Figure 3:**
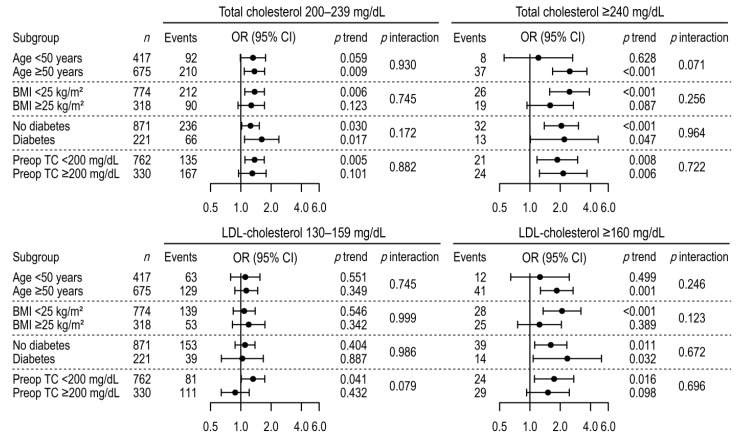
Association between follow-up TSH group and hypercholesterolemia in subgroup analysis. Multinomial multivariable logistic regressions were adjusted for age at follow-up, follow-up period, cigarette smoking status, body mass index, systolic blood pressure, fasting glucose level, and preoperative cholesterol level. CI, confidence interval; OR, odds ratio; LDL, low-density lipoprotein; TSH, thyroid-stimulating hormone.

At follow-up, the prevalence of hypercholesterolemia—defined by the levels of either total or LDL-cholesterol—gradually increased in the higher TSH groups (Figure 2). In the multinomial multivariable logistic regression, we observed significantly higher odds for hypercholesterolemia in association with higher TSH levels after adjusting for age, follow-up duration, preoperative total cholesterol levels, and other variables (Table 3). Compared to TSH levels of 0.03–0.3 mIU/L, those of 0.3–2 mIU/L were associated with 1.86 and 5.08 times higher odds for hypercholesterolemia (total cholesterol levels of 200–240 and ≥240 vs. <200 mg/dL) and 2.76 times the odds for hyper-LDL-cholesterolemia (LDL-cholesterol ≥160 vs. <130 mg/dL), respectively. Similarly, TSH levels of 2–5 mIU/dL were associated with 2.85- and 6.95-times higher odds for total cholesterol levels of 200–239 and ≥240 mg/dL, respectively; and 2.08 and 4.17 times higher odds for LDL-cholesterol levels of 130–159 and ≥160 mg/dL, respectively. There were significant linear trends of increasing odds for hypercholesterolemia toward the higher TSH group. The associations and trends were similar, with no significant heterogeneities, between the subgroups by age, BMI, diabetes, and preoperative total cholesterol level (Figure 3). Finally, when a binary logistic regression model was used for the sensitivity analysis, we observed associations between high TSH levels and hypercholesterolemia (total cholesterol level ≥200 and ≥240 mg/dL; LDL-cholesterol level ≥130 and ≥160 mg/dL) that were similar to those identified in the multinomial analyses (Table S1).

## 4. Discussion

In this study, thyroidectomized DTC patients with mildly suppressed TSH levels (0.03–0.3 mIU/L) at follow-up had total cholesterol levels that were comparable to those at the preoperative stage. Patients with normal TSH levels at follow-up had total cholesterol levels that were significantly higher than those at the preoperative stage. As a result, patients with normal TSH levels had a significantly higher risk for hypercholesterolemia (both total cholesterol and LDL-cholesterol) than those with mildly suppressed TSH levels.

TSH is the most sensitive marker for thyroid function evaluation, and thyroid function status is generally assessed based on a reference TSH range defined in a healthy population with normal thyroid function [12]. However, the concept that normal TSH levels is the therapeutic target for thyroid hormone replacement is recently challenged. In a large population study by Peterson et al, levothyroxine-treated participants with normal serum TSH levels were found to be taking statins more often than taking euthyroid controls [5]. Similarly, meta-analysis by McAninch et al. showed that levothyroxine-treated patients with normal serum TSH levels had higher serum total cholesterol and LDL-cholesterol levels than did healthy controls [6]. This epidemiological evidence suggests that normal TSH levels do not assure sufficient thyroid hormone replacement in patients with hypothyroidism and that serum cholesterol levels may serve as an ancillary marker for adequacy of the replacement therapy [13].

Similarly, some evidence suggests that normal TSH levels in thyroidectomized patients may correspond to insufficient replacement of thyroid hormone. In the previous studies, thyroidectomized patients had lower TSH levels when their free T3 levels are similar to those at the preoperative stage or those of controls with normal thyroid function [7,8,14,15]. More recently, Ito et al. compared the preoperative and postoperative serum levels of the peripheral markers of thyroid function (lipoproteins, sex hormone-binding globulin, and bone metabolic markers) in 133 patients with papillary thyroid carcinoma who underwent total thyroidectomy [7]. In that study, patients with mildly suppressed TSH levels (0.03–0.3 mIU/L) showed no changes in the metabolic marker levels from the preoperative levels, whereas patients with normal TSH levels (0.3–5 mIU/L) showed elevated LDL-cholesterol levels and decreased bone turnover marker levels [7].

Thyroid hormones influence the function of various factors associated with cholesterol metabolism, such as that of the LDL-cholesterol receptor, cholesterol ester transfer protein, hepatic lipase and lipoprotein lipase, and the flow of bile acid in the liver [16,17]. Thus, insufficient replacement of thyroid hormones in hypothyroidism may result in increased total and LDL-cholesterol levels, which, in turn, may lead to atherosclerotic cardiovascular diseases [13]. We found that patients with mildly suppressed TSH levels (0.03–0.3 mIU/L) had total cholesterol levels equivalent to those at the preoperative stage, in line with the findings of Ito et al. [7], while patients with lower-normal TSH levels (0.3–2 mIU/L) and upper-normal TSH levels (2–5 mIU/L) had progressively greater increases in their total cholesterol levels after thyroidectomy. Furthermore, patients with lower-normal TSH and upper-normal TSH levels had higher odds for hypercholesterolemia (adjusted odds ratios (AORs) for total cholesterol levels ≥240 mg/dL: 5.08 and 6.95, respectively) and hyper-LDL-cholesterolemia (AORs for LDL-cholesterol levels ≥160 mg/dL: 2.76 and 4.17, respectively) than those with mildly suppressed TSH levels (0.03–0.3 mIU/L). These results suggest that patients with TSH level maintenance within the normal range after total thyroidectomy may have a high risk of hypercholesterolemia induced by the physiologically insufficient replacement of the thyroid hormone.

As, in addition to insufficient replacement, over-replacement of thyroid hormones may also increase cardiovascular risk, it is important to balance the intensity of levothyroxine replacement therapy [18]. Several reports have stated that subclinical hyperthyroidism increases the risk for heart failure, atrial fibrillation, and cardiovascular disease-related and overall death [19]. However, unlike the results in subclinical hyperthyroidism, the occurrence rate of adverse cardiovascular outcomes is not known to increase when thyroidectomized patients or those receiving levothyroxine therapy have mildly suppressed TSH levels [20,21]. Flynn et al. assessed patients receiving levothyroxine replacement and reported that those with TSH levels < 0.04 mIU/L and TSH levels > 4 mIU/L had increased risks for cardiovascular diseases and arrhythmias compared to patients with TSH levels between 0.4 and 4 mIU/L [21]. However, the risk in patients with TSH levels between 0.04 and 0.4 mIU/L was not increased [21]. More recently, Hesselink et al. retrospectively assessed DTC patients who underwent total thyroidectomy [20]. In that study, patients with TSH levels <0.02 mIU/L showed higher cardiovascular mortality values than those with TSH levels >0.2 mIU/L, whereas patients with TSH levels between 0.02 and 0.2 mIU/L did not [20]. Our results, along with those of previous studies, suggest that a mildly suppressed TSH level (e.g., 0.03–0.3 mIU/L in our study) may be the metabolically neutral TSH level that is associated with low cardiovascular risk in thyroidectomized DTC patients receiving levothyroxine.

This study is unique and has strong clinical utility. To the best of our knowledge, this is the first study to show that normal TSH levels are associated with increased hypercholesterolemia risk in DTC patients receiving levothyroxine after total thyroidectomy. Although changes in the serum lipid levels during thyroxine withdrawal after total thyroidectomy have been assessed in some studies, the risk of hypercholesterolemia during levothyroxine therapy has not been [22,23,24,25]. Owing to the fact that thyroid cancers are detected early these days, leading to a low risk, and that most DTC patients have a favorable clinical course [26], the unsuppressed range of TSH may be applied to many such cases according to current management guidelines [3,4]. Therefore, hypercholesterolemia associated with insufficient replacement of the thyroid hormones may be a prevalent problem for many DTC patients today, whereas the problem has rarely been addressed before as more intensive TSH suppression therapy had been the common practice in the past.

Our study has some limitations that should be considered. First, normal aging may have contributed to the incidence of postoperative hypercholesterolemia observed in our study. However, based on our preoperative data, the slope of age-associated total cholesterol increment is estimated to be only 0.4 mg/dL per year of age. In the Korea National Health and Nutrition Examination Survey 2005, the slope of the age-cholesterol association in women was 1.2 mg/dL per year [27]. In this regard, it is unlikely that the attained age at follow-up significantly contributed to hypercholesterolemia incidence in our study. Second, our study was based on a single follow-up result after a relatively short follow-up period. Furthermore, hard cardiovascular outcomes such as myocardial infarction, stroke, and death were not assessed, although hypercholesterolemia is a major risk factor for cardiovascular disease [28]. Third, our results may not be directly generalizable to male patients, because of the potential interaction of sex with the relationship between thyroid function and lipid metabolism [23,29]. Fourth, as this was a single-center study mainly comprising middle-aged patients in Korea, the findings should be interpreted with caution when applied to patients with different genetic, demographic, or geographic backgrounds.

## 5. Conclusions

In conclusion, while the serum total cholesterol levels significantly increased when female DTC patients maintained normal TSH levels with levothyroxine after total thyroidectomy, the total cholesterol levels did not change in patients with mildly suppressed TSH levels. Accordingly, patients who retained normal TSH levels after thyroidectomy had a higher risk of hypercholesterolemia and hyper-LDL-cholesterolemia. Our study suggests that metabolic derangement risk owing to insufficient levothyroxine replacement should be considered when less-intense TSH suppression is required in postoperative DTC patients. Additionally, randomized control trials on the potential adverse outcomes associated with thyroid dysfunction are needed to determine the optimal target range of postoperative TSH in DTC patients after total thyroidectomy.

## Figures and Tables

**Table 1:** Descriptive statistics by TSH group.

Variables	TSH, mIU/L				*p* Value
	<0.03	0.03–0.3	0.3–2	2–5	
	(*n* = 473)	(*n* = 394)	(*n* = 164)	(*n* = 61)	
Age at surgery, years	50.6 ± 10	51.2 ± 11.2	47.6 ± 12	47.6 ± 12	0.001
Age at follow-up, years	52.7 ± 10.1	53.4 ± 11.2	50 ± 11.9	50.3 ± 11.8	0.002
Follow-up, years	2.2 ± 0.9	2.2 ± 0.9	2.3 ± 0.9	2.7 ± 1	<0.001
History of hypertension	145 (30.7%)	161 (40.9%)	48 (29.3%)	21 (34.4%)	0.007
History of diabetes	87 (18.4%)	79 (20.1%)	32 (19.5%)	10 (16.4%)	0.876
Ever smokers	26 (5.5%)	22 (5.6%)	11 (6.7%)	5 (8.2%)	0.805
Preoperative TSH ^*^	1.89 ± 1.02	1.99 ± 1.01	1.9 ± 0.99	1.79 ± 1.08	0.385
Preoperative cholesterol	184.83 ± 28.49	184.34 ± 27.82	178.74 ± 27.6	177.23 ± 27.3	0.028
Body mass index, kg/m²	23.44 ± 3.07	23.98 ± 3.49	23.82 ± 3.67	24.03 ± 3.57	0.101
Systolic blood pressure, mmHg	124.73 ± 14.99	125.08 ± 15.46	123.66 ± 16.05	122.07 ± 13.3	0.438
Free T4, ng/dL ^†^	1.55 ± 0.26	1.44 ± 0.32	1.31 ± 0.23	1.18 ± 0.23	<0.001
TSH, mIU/L	0.02 ± 0	0.09 ± 0.06	0.84 ± 0.45	3.16 ± 0.88	<0.001
Glucose, mg/dL	98.92 ± 19.58	101.29 ± 24.18	100.13 ± 20.71	94.7 ± 13.03	0.099
Total cholesterol, mg/dL	181.14 ± 30.79	184.47 ± 30.57	191.2 ± 32.44	193.69 ± 33.65	<0.001
Total cholesterol, mg/dL					0.001
<200	345 (72.9%)	269 (68.3%)	98 (59.8%)	33 (54.1%)	
200–239	113 (23.9%)	113 (28.7%)	53 (32.3%)	23 (37.7%)	
≥240	15 (3.2%)	12 (3.1%)	13 (7.9%)	5 (8.2%)	
∆Total cholesterol, mg/dL	−3.69 ± 28.85	0.13 ± 28.09	12.46 ± 29.84	16.46 ± 29.78	<0.001
Triglyceride, mg/dL	110.5 ± 56.54	114.8 ± 60.05	110.1 ± 61.73	132.18 ± 70.88	0.048
HDL-cholesterol, mg/dL	51.7 ± 11.72	51.2 ± 11.74	53.92 ± 11.24	51.49 ± 9.55	0.084
LDL-cholesterol, mg/dL	107.34 ± 28.4	110.32 ± 28.12	115.26 ± 29.44	115.76 ± 31.14	0.007
LDL-cholesterol, mg/dL					0.04
<130	376 (79.5%)	304 (77.2%)	126 (76.8%)	41 (67.2%)	
130–159	81 (17.1%)	73 (18.5%)	24 (14.6%)	14 (23%)	
≥160	16 (3.4%)	17 (4.3%)	14 (8.5%)	6 (9.8%)	

Data are presented as mean ± standard deviation or frequency (%). * Preoperative TSH level data available in 929 patients. ^†^ Follow-up T4 level data available in 1009 patients. Abbreviations: HDL, high-density lipoprotein; LDL, low-density lipoprotein; TSH, thyroid-stimulating hormone.

**Table 2:** Association between follow-up TSH level and ∆total cholesterol (mg/dL).

Model	*n*	β (SE)	*p* Value
Model 1	1092	6.45 (1.11)	<0.001
Model 2	1092	5.15 (1.03)	<0.001
Model 3	1092	5.15 (1.03)	<0.001

Coefficients are in mg/dL per 1 mIU/L increment in TSH level. Model 1 was unadjusted. Model 2 was adjusted for age at follow-up, duration of follow-up, and preoperative cholesterol level. Model 3 was further adjusted for cigarette smoking status, BMI, SBP, and fasting glucose level. Abbreviations: BMI, body mass index; SBP, systolic blood pressure; SE, standard error; TSH, thyroid-stimulating hormone.

**Table 3:** Association between follow-up TSH level and hypercholesterolemia.

**Model**	**TSH, mIU/L**	***n***	**Total Cholesterol Level 200–239 mg/dL**				**Total Cholesterol Level ≥240 mg/dL**			
			**Events**	**OR (95% CI)**	***p* Value**	***p* for Trend**	**Events**	**OR (95% CI)**	***p* Value**	***p* for Trend**
**Model 1**	<0.03	473	113	0.78 (0.57–1.06)	0.111		15	0.97 (0.45–2.12)	0.948	
	0.03–0.3	394	113	1 (reference)			12	1 (reference)		
	0.3–2	164	53	1.29 (0.86–1.92)	0.216		13	2.97 (1.31–6.74)	0.009	
	2–5	61	23	1.66 (0.93–2.95)	0.085	0.102	5	3.4 (1.13–10.25)	0.03	0.003
**Model 2**	<0.03	473	113	0.73 (0.52–1.02)	0.064		15	0.93 (0.42–2.07)	0.854	
	0.03–0.3	394	113	1 (reference)			12	1 (reference)		
	0.3–2	164	53	1.84 (1.18–2.87)	0.008		13	4.9 (2.05–11.72)	<0.001	
	2–5	61	23	2.61 (1.37–4.98)	0.004	0.002	5	6.21 (1.89–20.34)	0.003	<0.001
**Model 3**	<0.03	473	113	0.74 (0.52–1.03)	0.078		15	1.04 (0.46–2.32)	0.930	
	0.03–0.3	394	113	1 (reference)			12	1 (reference)		
	0.3–2	164	53	1.86 (1.19–2.92)	0.007		13	5.08 (2.14–12.06)	<0.001	
	2–5	61	23	2.85 (1.47–5.53)	0.002	0.001	5	6.95 (2.13–22.72)	0.001	<0.001
**Model**	**TSH, mIU/L**	***n***	**LDL-Cholesterol Level 130–159 mg/dL**				**LDL-Cholesterol Level ≥160 mg/dL**			
			**Events**	**OR (95% CI)**	***p* Value**	***p* for Trend**	**Events**	**OR (95% CI)**	***p* Value**	***p* for Trend**
**Model 1**	<0.03	473	81	0.9 (0.63–1.27)	0.544		16	0.76 (0.38–1.53)	0.444	
	0.03–0.3	394	73	1 (reference)			17	1 (reference)		
	0.3–2	164	24	0.79 (0.48–1.32)	0.369		14	1.99 (0.95–4.15)	0.068	
	2–5	61	14	1.42 (0.74–2.75)	0.295	0.953	6	2.62 (0.98–7.02)	0.056	0.02
**Model 2**	<0.03	473	81	0.84 (0.58–1.23)	0.377		16	0.73 (0.36–1.5)	0.396	
	0.03–0.3	394	73	1 (reference)			17	1 (reference)		
	0.3–2	164	24	0.99 (0.58–1.7)	0.972		14	2.66 (1.22–5.78)	0.014	
	2–5	61	14	2.04 (0.99–4.18)	0.053	0.306	6	3.9 (1.36–11.19)	0.012	0.003
**Model 3**	<0.03	473	81	0.84 (0.57–1.22)	0.357		16	0.85 (0.41–1.78)	0.668	
	0.03–0.3	394	73	1 (reference)			17	1 (reference)		
	0.3–2	164	24	1 (0.58–1.71)	0.991		14	2.76 (1.24–6.12)	0.012	
	2–5	61	14	2.08 (1.01–4.31)	0.048	0.298	6	4.17 (1.41–12.34)	0.01	0.002

Model 1 was unadjusted. Model 2 was adjusted for age at follow-up, duration of follow-up, and preoperative cholesterol level. Model 3 was further adjusted for cigarette smoking status, body mass index, systolic blood pressure, and fasting glucose level. Abbreviations: CI, confidence interval; OR, odds ratio; LDL, low-density lipoprotein; TSH, thyroid-stimulating hormone.

## Supplementary Materials

The following are available online at https://www.mdpi.com/2077-0383/8/8/1106/s1, Figure S1. Flowchart of the study population; Table S1. Association between follow-up TSH level and hypercholesterolemia examined using conventional binary logistic regression.
