# Supplementary material for: Association between Thyroid-Stimulating Hormone Level after Total Thyroidectomy and Hypercholesterolemia in Female Patients with Differentiated Thyroid Cancer: A Retrospective Study
Source: J Clin Med. 2019 Jul 25;8(8):1106. doi: 10.3390/jcm8081106 (PMC6723726; doi:10.3390/jcm8081106)
Supplement: Supplementary file 1 [file jcm-08-01106-s001.pdf]

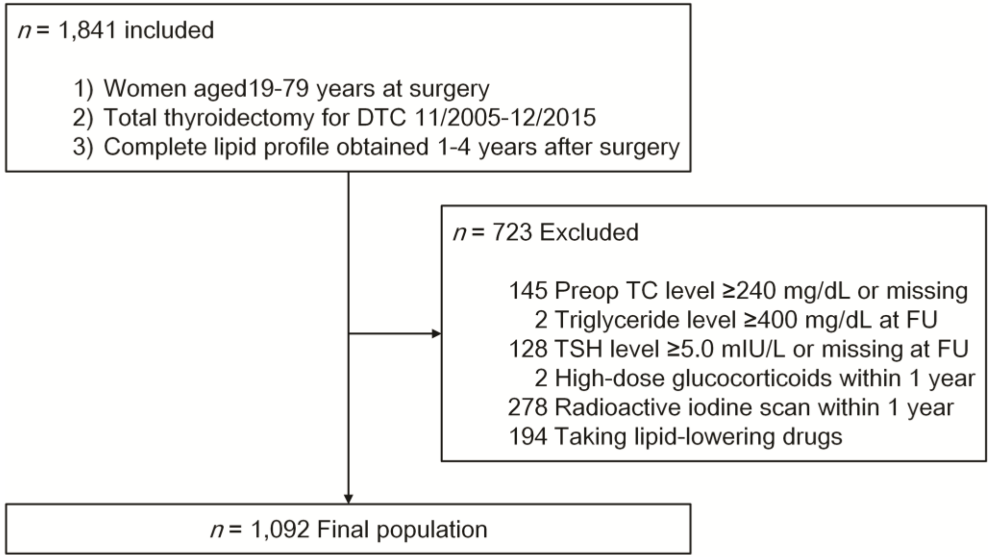

Figure S1. Flowchart of the study population. DTC, differentiated thyroid carcinoma; TC, total cholesterol; TSH, thyroid-stimulating hormone; FU, follow-up.

Table S1. Association between follow-up TSH level and hypercholesterolemia examined using conventional binary logistic regression.

**Model**	**TSH, mIU/L**	*n*	Total cholesterol level ≥200 mg/dL				Total cholesterol level ≥240 mg/dL			
			Events	OR (95% CI)	*p* value	*p* for trend	Events	OR (95% CI)	*p* value	*p* for trend
**Model 1**	**<0.03**	473	128	0.8 (0.6–1.07)	0.133		15	1.04 (0.48–2.25)	0.916	
	0.03–0.3	394	125	1 (reference)			12	1 (reference)		
	0.3–2	164	66	1.45 (0.99–2.11)	0.054		13	2.74 (1.22–6.14)	0.014	
	2–5	61	28	1.83 (1.06–3.15)	0.031	0.017	5	2.84 (0.96–8.37)	0.058	0.006
Model 2	<0.03	473	128	0.75 (0.54–1.03)	0.077		15	1.06 (0.48–2.32)	0.89	
	0.03–0.3	394	125	1 (reference)			12	1 (reference)		
	0.3–2	164	66	2.08 (1.35–3.19)	<0.001		13	3.67 (1.58–8.49)	0.002	
	2–5	61	28	2.89 (1.55–5.39)	<0.001	<0.001	5	3.92 (1.27–12.17)	0.018	<0.001
Model 3	<0.03	473	128	0.76 (0.55–1.06)	0.104		15	1.19 (0.53–2.64)	0.678	
	0.03–0.3	394	125	1 (reference)			12	1 (reference)		
	0.3–2	164	66	2.11 (1.36–3.25)	<0.001		13	3.78 (1.61–8.86)	0.002	
	2–5	61	28	3.17 (1.67–6)	<0.001	<0.001	5	4.16 (1.31–13.22)	0.016	<0.001

Model	TSH, mIU/L	*n*	LDL–cholesterol level ≥130 mg/dL				LDL–cholesterol level ≥160 mg/dL			
			Events	OR (95% CI)	*p* value	*p* for trend	Events	OR (95% CI)	*p* value	*p* for trend
Model 1	<0.03	473	97	0.87 (0.63–1.21)	0.405		16	0.78 (0.39–1.56)	0.476	
	0.03–0.3	394	90	1 (reference)			17	1 (reference)		
	0.3–2	164	38	1.02 (0.66–1.57)	0.933		14	2.07 (1–4.3)	0.052	
	2–5	61	20	1.65 (0.92–2.95)	0.094	0.298	6	2.42 (0.91–6.4)	0.075	0.02
Model 2	<0.03	473	97	0.82 (0.58–1.17)	0.282		16	0.76 (0.37–1.56)	0.457	
	0.03–0.3	394	90	1 (reference)			17	1 (reference)		
	0.3–2	164	38	1.28 (0.8–2.06)	0.308		14	2.66 (1.24–5.7)	0.012	
	2–5	61	20	2.37 (1.23–4.54)	0.01	0.039	6	3.14 (1.13–8.74)	0.028	0.004
Model 3	<0.03	473	97	0.84 (0.59–1.19)	0.326		16	0.89 (0.43–1.84)	0.756	
	0.03–0.3	394	90	1 (reference)			17	1 (reference)		
	0.3–2	164	38	1.28 (0.8–2.06)	0.305		14	2.76 (1.26–6.04)	0.011	
	2–5	61	20	2.44 (1.26–4.71)	0.008	0.035	6	3.36 (1.17–9.63)	0.024	0.003

Binary multivariable logistic regression model was adjusted for age at follow-up, duration of follow-up, cigarette smoking status, body mass index, systolic blood pressure, fasting glucose level, and preoperative cholesterol level. Abbreviations: CI, confidence interval; OR, odds ratio; LDL, low-density lipoprotein; TSH, thyroid-stimulating hormone.
